# The hyperfunction theory of aging: three common misconceptions

**DOI:** 10.18632/oncoscience.545

**Published:** 2021-09-17

**Authors:** Mikhail V. Blagosklonny

**Affiliations:** ^1^Roswell Park Cancer Institute, Buffalo, NY 14263, USA

**Keywords:** geroscience, rapamycin, mTOR, geroconversion, quasi-program, cell senescence

Almost all papers on aging start with the statement that aging is functional decline caused by accumulation of molecular damage. In contrast, according to the hyperfunction theory, aging is not functional decline, but results from cellular hyperfunctions that produce age-related diseases. The sum of these age-related diseases is aging (Figure 1). Functional decline is secondary to primary hyperfunctions. Second, aging is not caused by the accumulation of molecular damage—it is caused by inappropriate activation of signaling pathways, such as mTOR. These signaling pathways directly drive age-related diseases. The hyperfunction theory of aging was discussed in detail in numerous papers (just to name a few [1–10]). However, given the unconventional nature of the hyperfunction theory, some points are commonly misunderstood. Here, I will address three common misconceptions people tend to have about the hyperfunction theory, without discussing the theory in detail.

**Figure 1:**
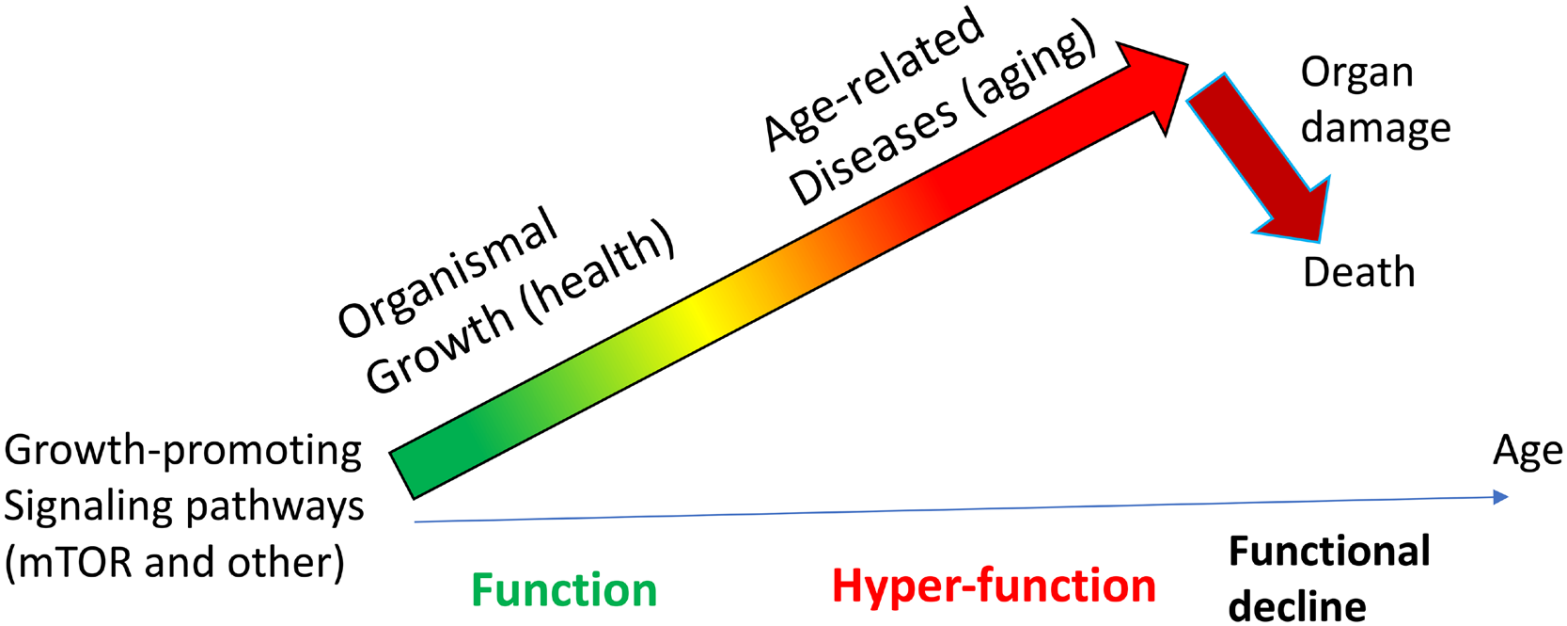
General presentation of hyperfunction theory. Aging is a hyper-function caused by unnecessary and persistently activated signaling pathways, such as mTOR (for example), not by molecular damage. These signaling pathways activate cells, directly causing the development of age-related diseases. Aging is a sum of all diseases. Hyperfunctions may eventually lead to organ damage and loss of functions. Adopted with modifications from Figure 1 in ref. [1].

## MISCONCEPTION 1

### Hyperfunction is always an increase of function. Correctly, hyperfunction is often an unchanged function, that is still higher than optimal for longevity

**Figure 2:**
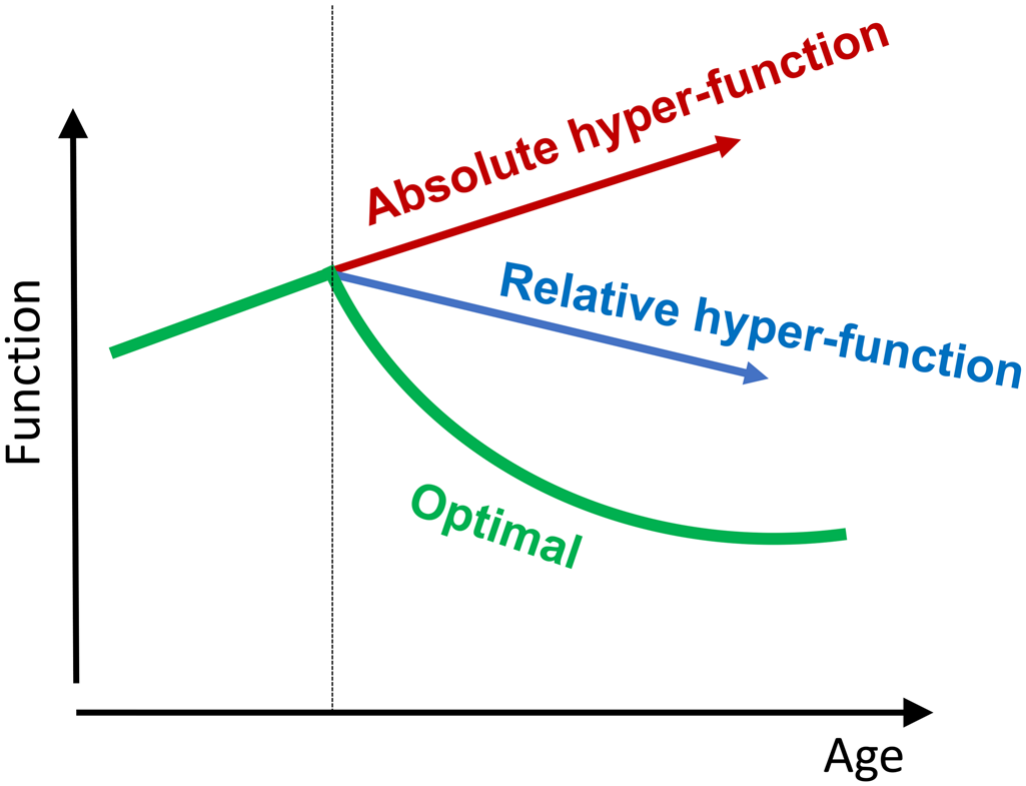
Absolute and relative hyperfunctions. Optimal function is age-dependent.

Hyperfunction is a function that was not switched off upon its completion [1]. In some cases, age-related alterations are indeed an absolute increase: hyper-secretory phenotype, pro-inflammation, hypertension, hyperlipidemia, hyperglycemia, hyperinsulinemia, hyperplasia and hypertrophy of cells and organs (e.g., heart and prostate). In typical cases, hyperfunction is relative. It may even be a decrease of function that is still higher than optimal for longevity in the aging organism (Figure 2).

Using an analogy, consider a car driving 65 miles per hour (mph) on the highway with a 65 mph speed limit. This is the normal and optimal speed on this highway, or optimal functioning early in life. Early in life, during organism growth, all cellular and systemic functions are optimal for growth (not for longevity). However, if the car exits the highway to enter low-speed streets without decreasing speed (stuck accelerator) and continues at the same speed, 65 mph becomes over-speeding, or hyperfunction. The car is bound to crash on your driveway and is destroyed by over-speeding. It has no chance to be destroyed on a molecular level by rusting. Similarly, hyperfunction causes organ damage (e.g. stroke, infarction, cancer metastasis, broken hip) and death before molecular damage (rusting by free radicals) accumulates to deadly levels [2].

## MISCONCEPTION 2

### Hyperfunction vs. molecular damage

**Figure 3:**
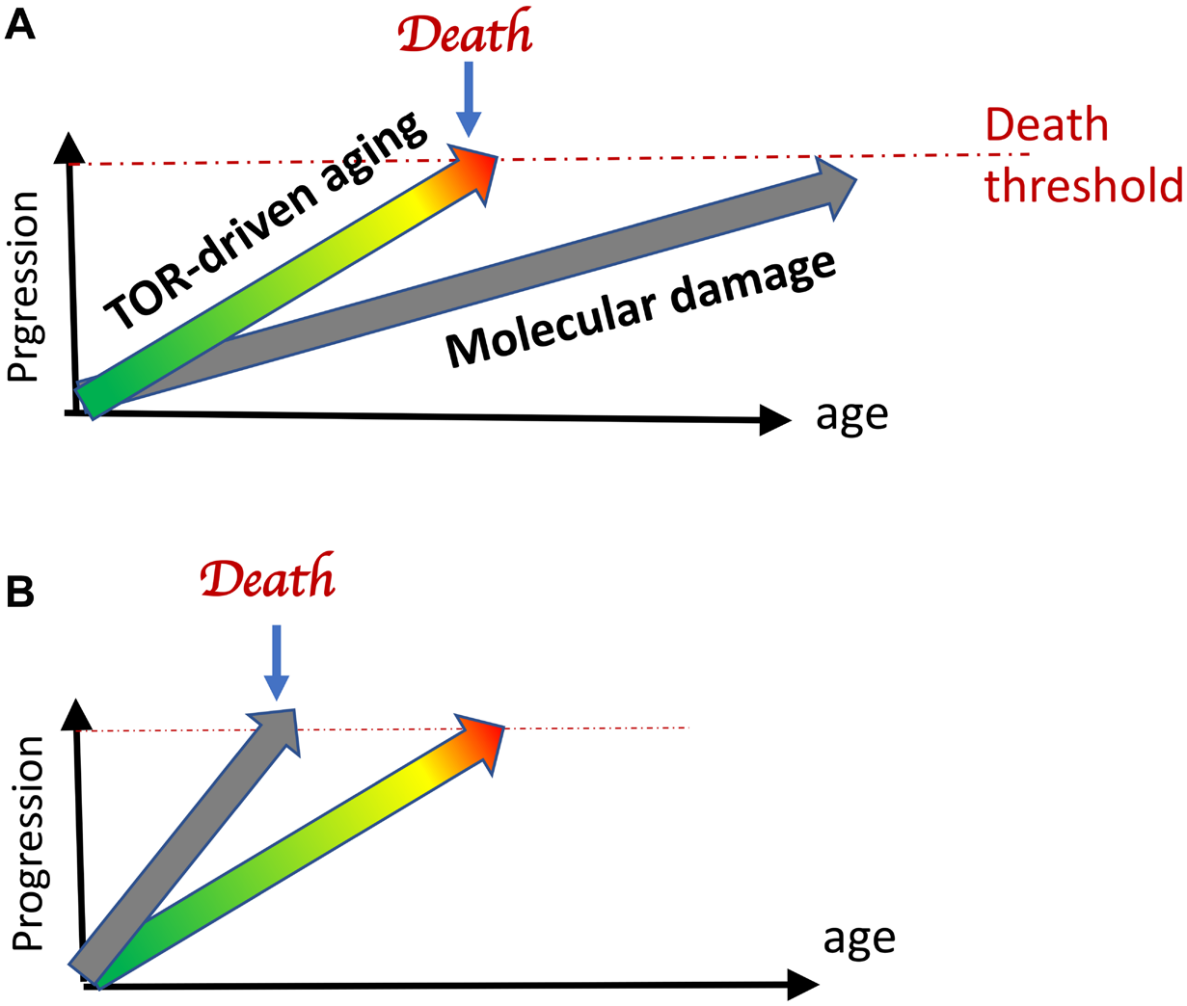
Life-limiting aging. (**A**) Normal Aging. Hyperfunctional (mTOR-driven) aging is life-limiting. It reaches a deadly threshold earlier than accumulating molecular damage does. (**B**) Premature aging syndromes. When artificially accelerated by gene knockouts, accumulation of molecular damage may become life-limiting. Adopted from ref. [10]).

The second misconception is that the hyperfunction theory of aging denies a harmful accumulation of molecular damage. To clarify, molecular damage does accumulate. Furthermore, molecular damage would eventually kill the organism, unless the organism dies from hyperfunctional aging or, even more specifically, from mTOR-driven aging (Figure 3). Aging due to molecular damage and due to cellular hyperfunctions occur in parallel, but the latter is a life-limiting process, which progresses faster.

How do we know that hyperfunctional aging is life-limiting and accumulation of molecular damage is not? In several dozen studies, rapamycin (mTORC1 inhibitor) prolonged lifespan in animals (see for references [10, 11]). In some short-lived mice, rapamycin even tripled maximal lifespan [12]. Then mTOR-driven aging is life-limiting almost by definition.

In contrast, a decrease of molecular damage does not prolong lifespan in most studies. Interestingly, a very mild increase of molecular damage may prolong lifespan, probably by inhibiting mTOR [13]. To make molecular damage life-limiting, it is necessary to dramatically increase it by knocking out key enzymes, telomerases, and so on [10]. Of note, symptoms of molecular damage-induced premature aging are different from normal age-related diseases. In addition, hyperfunctional aging must be life-limiting from a theoretical perspective, and it was predicted in 2006 (before rapamycin was tested in animals) that rapamycin must extend lifespan in animals and humans [1].

## MISCONCEPTION 3

### The hyperfunction theory is primarily based on an evolutionary theory. Correctly, the hyperfunction theory is principally based on a cellular model of geroconversion

The hyperfunction theory is not just an evolutionary theory, even though it is completely in agreement with the latter and develops the notion of Antagonistic Pleiotropy (AP) further. Evolutionary perspectives in the hyperfunction theory are needed mostly to explain why hyperfunctional (quasi-programmed) aging is life-limiting and why accumulation of molecular damage is not [9]. (Note: Hyperfunction/quasi-program is an aimless and harmful continuation of a program that was not switched off upon its completion.)

**Figure 4:**
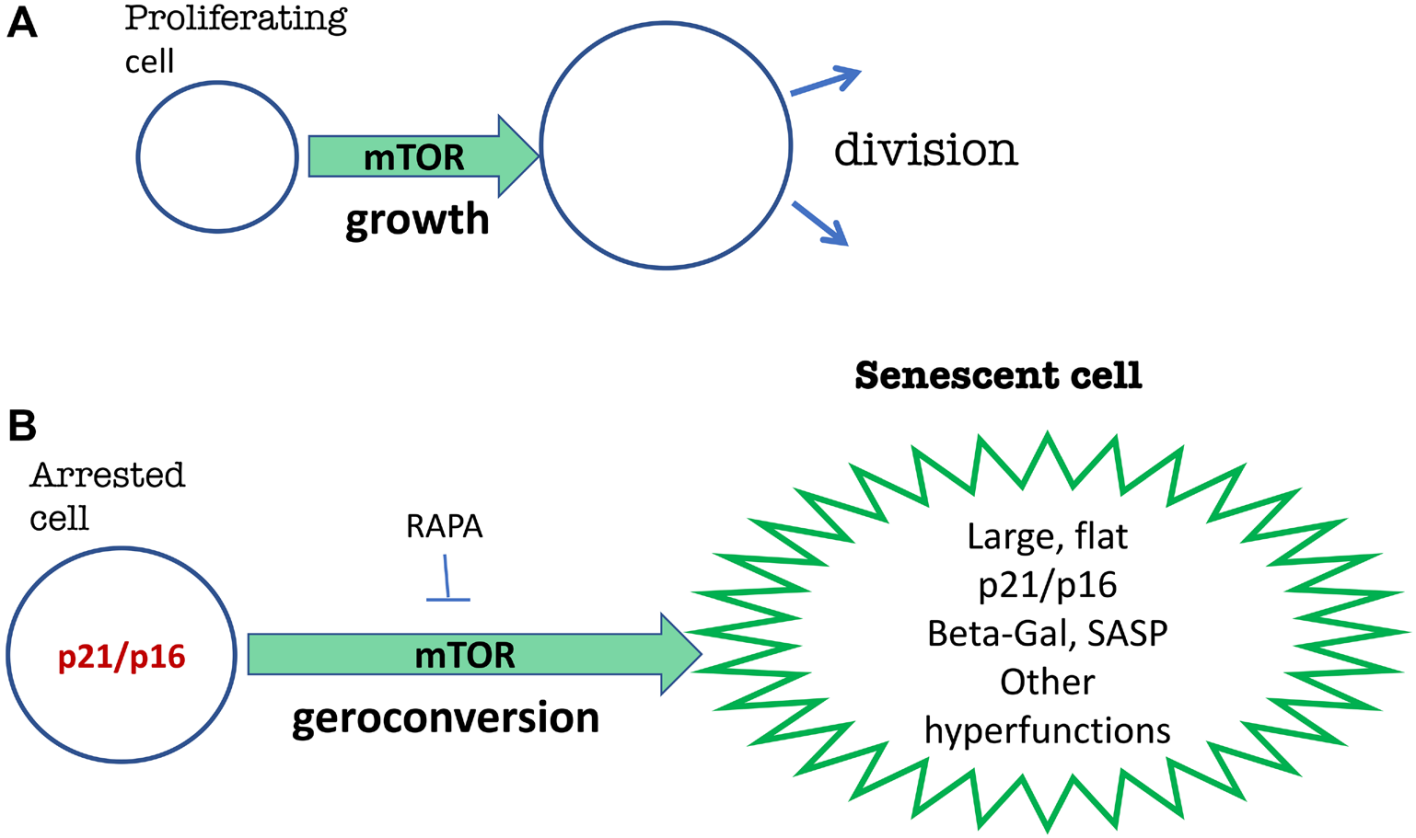
Geroconversion model (in cell culture) displays basic features of hyperfunction theory. (**A**) In proliferating cells, growth-promoting pathways such as mTOR drive cell mass growth, which is balanced by cell division. (**B**) When the cell cycle is suddenly blocked by p21 and p16, growth-promoting pathways such as mTOR drive geroconversion to senescence. Proliferation-like activity of mTOR in non-proliferating cells is a hyperfunction. Senescent cells display various hyperfunctions on a cellular level. See text for details.

Otherwise, the hyperfunction theory is a mechanistic theory: an analogy of the cellular model of geroconversion *in vitro* (Figure 4). When cells proliferate, mTOR and other growth-promoting signaling pathways drive cellular mass growth, which is balanced by cell division. However, if the cell cycle is blocked by p21 or p16, then the same mTOR pathway drives “pathological growth” (geroconversion) from reversible arrest to irreversible senescence [14]. Geroconversion is a continuation of growth—a quasi-program of growth. The arrested cells grow in size exponentially [15] (without division) and become large, flat, and hyperfunctional, namely beta Gal-positive (lysosomal hyperfunction), SASP (hyper-secretory phenotype), and compensatory insulin/growth factor-resistant [16, 17], as could be predicted on theoretical grounds [18]. The hyperfunction theory was derived from cancer research because the same signaling pathways are involved in both cancer and geroconversion.

The hyperfunction theory is a translation of the rules of geroconversion to the organism. Organismal aging and geroconversion can be described in similar terms, and similar signaling pathways drive geroconversion and organismal aging [1].

It does not necessarily mean that a few senescent cells cause organismal aging. Fully senescent cells may contribute to aging, but are not required [19]. Instead, most cells are becoming at least relatively hyperfunctional, gerogenic, producing age-related diseases, as exemplified by the development of atherosclerosis and hypertension, which may culminate in infarction and death [20]. The geroconversion model is a simplified model of quasi-programmed or hyperfunctional organismal aging.

On an organismal level, the hyperfunction theory describes the development of age-related diseases [20]. David Gems and co-workers demonstrated that age-related diseases in *C. elegans* are quasi-programmed, developing a fruitful model for the hyperfunction theory [8, 21–23]. Even aging in yeast remotely models the hyperfunction theory, and mTOR was identified as one of drivers of yeast aging [24, 25].

Geroconversion is driven by continuously active growth-promoting signaling pathways such as PI3K/mTOR and MEK/MAPK, which are maximally activated in proliferating cells and continue to be active through external signaling (e.g., insulin, nutrients, GFs), which establishes positive feedback loops in the organism. Geroconversion is not driven by the genome, although gene expression changes dramatically during geroconversion, partially ensuring its irreversibility.

The idea that aging is a continuation of development is not new [26–28] and, as a general notion, was entertained even before the term gerontology was coined [28]. As put by Walker, who revisited developmental theories, “developmental inertia is the primary cause of senescence”. [29] Without specific mechanisms, the idea is too general or vague to have clinical applications. The developmental theory of aging by Dilman implicated one organ—the hypothalamus (a tiny part of the brain) [26, 27]. I discussed this developmental theory of aging in the light of the hyperfunction theory [30].

The essence of hyperfunction theory is that, later in life, higher than optimal activity of signal-transduction pathways (maintained by positive feedback loops) directly drives age-related diseases (which are, in sum, aging). This notion is most appealing to practicing physicians, who have implemented this theory in the treatment of age-related diseases by slowing aging https://rapamycintherapy.com/.

In sum, the hyperfunction theory of organismal aging was initially developed as an analogy to the geroconversion model. The geroconversion model makes it possible to discover anti-aging drugs (Figure 4), because drugs that inhibit geroconversion slow down organismal aging. Rapamycin slows geroconversion, predicting that rapamycin would increase the lifespan of animals. Therefore, in 2006, it was suggested that rapamycin (clinically available since 1999) can be used in humans immediately to slow down aging and all age-related diseases [1].
